# Influence of the electrocatalyst layer thickness on alkaline DEFC performance†

**DOI:** 10.1039/d2se01729f

**Published:** 2023-01-27

**Authors:** Michaela Roschger, Sigrid Wolf, Kurt Mayer, Andreas Billiani, Boštjan Genorio, Selestina Gorgieva, Viktor Hacker

**Affiliations:** a Institute of Chemical Engineering and Environmental Technology, Graz University of Technology Inffeldgasse 25/C 8010 Graz Austria michaela.roschger@tugraz.at; b Faculty of Chemistry and Chemical Technology, University of Ljubljana Večna pot 113 1000 Ljubljana Slovenia; c Faculty of Mechanical Engineering, University of Maribor Smetanova ulica 17 2000 Maribor Slovenia

## Abstract

Determining the optimum layer thickness, for the anode and cathode, is of utmost importance for minimizing the costs of the alkaline direct ethanol fuel cell (DEFC) without lowering the electrochemical performance. In this study, the influence of layer thickness on the performance of the ethanol oxidation reaction (EOR) and oxygen reduction reaction (ORR) in an alkaline medium and resistance was investigated. The prepared gas diffusion electrodes (GDEs) were fully characterized, with scanning electron microscopy to determine the layer thickness and electrochemically in half-cell configuration. Cyclic voltammetry and polarization curve measurements were used to determine the oxidation and reduction processes of the metals, the electrochemical active surface area, and the activity towards the ORR and EOR. It was demonstrated that realistic reaction conditions can be achieved with simple and fast half-cell GDE measurements. Single cell measurements were conducted to evaluate the influence of factors, such as membrane or ethanol crossover. In addition, electrochemical impedance spectra investigation was performed to identify the effect of layer thickness on resistance. This successfully demonstrated that the optimal layer thicknesses and high maximum power density values (120 mW cm^−2^) were achieved with the Pt-free catalysts and membranes used.

## Abstract 2

The optimum layer thickness of Ag-Mn_*x*_O_*y*_/C and PdNiBi/C electrodes for use in alkaline direct ethanol fuel cells at a high power density of 120 mW cm^−2^ and its influence on resistance were determined.

## Graphical abstract

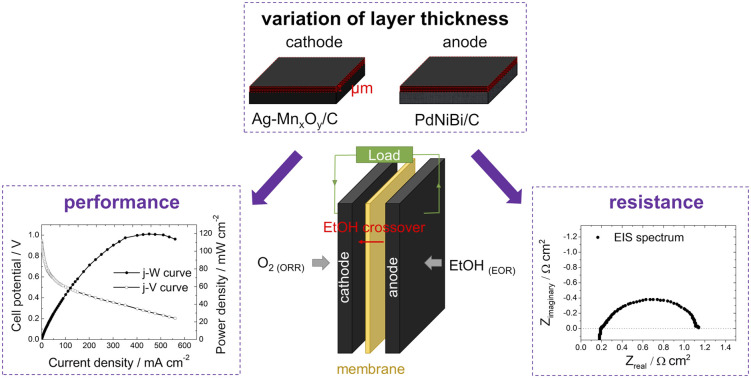

## Introduction

Alkaline direct ethanol fuel cells (DEFCs) are promising energy conversion devices in which chemical energy stored in ethanol is converted directly into electrical energy through electrochemical reactions at the anode and cathode. This makes DEFCs a sustainable technology because ethanol can be produced renewably and has a high energy density. In addition, ethanol is a liquid fuel that is safe to handle and easy to transport. Furthermore, the caustic conditions of alkaline DEFCs bring other advantages such as better reaction kinetics and the use of non-precious metal catalysts at the cathode, where the oxygen reduction reaction (ORR) takes place. In an alkaline DEFC, oxygen is reduced at the cathode and produces H_2_O and OH^−^ ions.^1–7^ In recent years, much research has been conducted in the field of these catalysts and low-cost alternatives have been found instead of expensive Pt catalysts. Many studies have been conducted on Au or Ag-based catalysts, as well as spinels, perovskites, manganese oxides and a variety of dopants.^8–12^ In addition to performance and cost reduction, another important factor in an alkaline DEFC is ethanol tolerance. Since ethanol can diffuse through the membrane and react with employed Pt/C catalysts on the cathode, mixed potentials are formed, leading to a reduction in power.^2,9,13^ It has been shown in the literature that cheaper silver-manganese oxide catalysts also have better ethanol tolerance than Pt.^11,12^

In addition to the enhanced reaction kinetics, the alkaline medium enables the use of cost-effective and environmentally friendly anion exchange membranes (AEMs), which are responsible for the conduction of OH^−^ ions from the cathode to the anode. However, commercially available AEMs mostly consist of expensive and synthetic polymers, despite the fact that there are already studies in the literature on the utilization of manufacturing AEMs with bio-based polymers. One such option is the use of chitosan (exoskeletons of crustaceans), which is also thermally stable and non-toxic. Through attachment of quaternary ammonium groups, for improved anion conduction, since chitosan has poor conductivity, and the addition of graphene materials, well-conducting and stable (chemically, thermally and mechanically) membranes can be created.^1,2,8,14,15^

Despite the many advantages of the alkaline medium, the ethanol oxidation reaction (EOR) in which ethanol is converted with OH^−^ ions is hindered. Currently, neither Pt nor the more effective Pd catalysts in an alkaline medium are able to completely convert ethanol to CO_2_ due to C–C bond breaking issues. Products such as acetate, acetic acid or acetaldehyde are formed.^1,3,4,6^ Studies in the literature have shown that by adding atoms (Ag, Au, Ni, Bi, Sn, Ru, Cu, and Pb) to Pd-based catalysts the EOR performance can be improved.^2,13^ Ni or Bi, for example, which are oxophilic elements, contribute to the improvement by facilitating the oxidation process and the oxidative removal of poisonous intermediate species.^16–18^ Since the catalysts are not capable of completely converting ethanol, the alkaline DEFC is currently operated as a liquid-feed cell, which means that KOH is added to the fuel mixture on the anode side (more OH^−^ available). The use of a liquid electrolyte in addition to a membrane involves disadvantages such as carbonation. Possibly generated CO_2_ in the cell can react with KOH to form carbonates, which then block the pores of the catalysts.^1^ However, the addition of the electrolyte to the fuel supports the EOR and compensates for the poorer ion conduction (H^+^ mobility is much better than OH^−^) of AEMs compared to cation exchange membranes at the moment.^1,2,5,8,19^

Complementary to the study on the optimization of catalysts, the overall cell output can be further improved by researching the electrode geometry made out of them and the processing, *e.g.* the catalyst layer utilization and the transport of reactants that influence the performance. One possibility for improvement and cost reduction is the reduction of the catalyst loading and the associated reduction of the layer thickness. Reducing the metal loading reduces the costs as, for example, the Pd catalyst is a driving factor of the cell price. However, layers on the anode side that are too thin can promote ethanol crossover.^20,21^ Research on the reduction of electrode layer thicknesses without PGM-catalysts is also important for achieving the best possible performance, despite the fact that these are not expensive.^22,23^ Layers that are too thick can reduce the transport of reactants, due to increased mass transfer resistances and thus not all of the active sites of the catalyst are utilized.^24–31^

In terms of determining the appropriate layer thickness for the anode and cathode, it is possible to determine the influences by means of half-cell measurements with the use of gas diffusion electrodes (GDEs).^32,33^ Compared to the single cell, only one electrode needs to be used per measurement and no membrane is required. It is therefore a cheaper research alternative to the single cell and for the determination of basic properties such as activity, resistance and onset potential of the electrodes. Moreover, the influencing factors, such as the membrane, are consequently negligible. Compared to conventional rotating disk electrode (RDE) measurements, GDE half-cell measurements show more realistic conditions, due to the presence of a three phase boundary and also the possibility to measure at high currents.^9,32–40^ RDE measurements, however, are extremely important for basic fundamental catalyst research, such as the oxidation and reduction processes, and useful by coupling with *in situ* Fourier-transform infrared spectroscopy (FT-IR) or Raman spectroscopy to study ethanol conversion and intermediates that accumulate on the catalyst.^41–44^ The influence of ethanol crossover, which has a large impact, as already described, can be achieved through the measurement of the entire fuel cell. During these measurements, the crossover, resulting products or ethanol consumption have been determined with ion exchange chromatography (IC), gas chromatography (GC) or FT-Raman measurements in the literature.^45–51^

In this study, the influence of layer thickness on the performance of the EOR and ORR in an alkaline medium and resistance was investigated. For this purpose, half-cell measurements of varying anode and cathode thicknesses were performed and the influence on activity and electrochemical active surface area (ECSA) was investigated. Furthermore, the electrodes were measured in single cell tests and the ethanol conversion was determined at varying anode thicknesses. Another important aspect was the influence of layer thickness on ethanol crossover and performance in a single cell. High maximum power density values, comparable to those reported in the literature,^52,53^ were achieved with the in-house made catalysts and membranes.

## Experimental

### Chemicals and materials

The following chemicals were used for the synthesis of the anode and cathode catalysts: Vulcan XC72R carbon black (CABOT Corporation), palladium chloride (PdCl_2_, anhydrous, 59–60% Pd basis, Aldrich), nickel(ii) nitrate hexahydrate (Ni(NO_3_)_2_·6H_2_O, 99% trace metal basis, Aldrich), bismuth(iii) chloride (BiCl_3_, reagent grade, ≥98%, Aldrich), hydrochloric acid (HCl, ROTIPURAN® 37% fuming, p.a., ACS, ISO, Carl Roth), sodium borohydride (NaBH_4_, purity of 97%, Alfa Aesar), sodium hydroxide (NaOH, ≥98%, ACS, pellets, Fluka), ultrapure water (∼18 MΩ cm, Barnstead NANOpureWater Purification system), potassium permanganate (KMnO_4_, ≥99.0% p.a., Merck), and silver nitrate (AgNO_3_, ≥99.8% p.a., Merck).

The following materials and chemicals were utilized for the MEA production and the half and single cell tests: ethanol (EtOH, 99.9% p.a., Roth), 2-propanol (99.9% p.a., Roth), potassium hydroxide (KOH, ≥85%, p.a., pellets), CS/N-rGONRs anion exchange membranes developed in our previous work,^14^ Nafion™ solution (NS-5, PTSA 5%, Quintech), commercial Pt/C (platinum, nominally 40% on carbon black, Alfa Aesar), carbon cloth (ELAT—hydrophilic plain cloth, fuel cell store, 0.406 mm thick), and carbon paper (Sigracet 29 BC, fuel cell store, 0.235 mm thick).

### Catalyst synthesis

#### Anode

The Pd_85_Ni_10_Bi_5_/C anode catalyst (30 wt% active material) was synthesized as described in the previously published literature.^18^ As no modifications were made to the synthesis of the catalyst, the synthesis itself is shown here in a very simplified form. Vulcan XC72R is dispersed in ultrapure water and then the three metal salt precursor solutions (PdCl_2_, Ni(NO_3_)_2_·6H_2_O and BiCl_3_ in water and HCl) are added. For the reduction process, the pH is adjusted to 10 and an alkaline solution of NaBH_4_ is added dropwise. After stirring the solution at 60 °C for four hours, filtration and drying at 40 °C, the catalyst material is obtained.

#### Cathode

The Ag-Mn_*x*_O_*y*_/C cathode catalyst (30 wt% active material) was prepared as stated in (ref. 12), with the exception that Vulcan XC72R (as it is a commercial material and widely studied^54^) was used as the support material. Therefore, the synthesis is again only briefly described here. First, Vulcan XC72R was ultrasonicated in a solution of ultrapure water and isopropanol. After addition of AgNO_3_ and KMnO_4_, the dispersion was sonicated and then stirred at 80 °C under reflux, followed by ultrasonication again. The prepared catalyst was obtained by filtration with water and subsequent drying at 40 °C.

### Electrode and membrane electrode assembly preparation

The as prepared anode and cathode catalysts were used to prepare the electrodes and membrane electrode assemblies (MEAs) for the evaluation of the influence of the layer thickness *via* half and single cell tests. A list of the different anode electrodes (A1–A4), cathode electrodes (C1–C5) and MEAs (MEA1–MEA8) is given in Table 1.

**Table 1:** List of the prepared electrodes and membrane electrode assemblies (MEAs)

Anodes	Catalyst	Loading^a^	Cathodes	Catalyst	Loading^a^
Electrodes					
A1	Pd_85_Ni_10_Bi_5_/C	0.25	C1	Ag-Mn_*x*_O_*y*_/C	0.25
A2		0.5	C2		0.5
A3		0.75	C3		0.75
A4		1	C4		1
			C5	Pt/C	0.5
MEAs					
MEA1	A1 + C5		MEA5	A2 + C1	
MEA2	A2 + C5		MEA6	A2 + C2	
MEA3	A3 + C5		MEA7	A2 + C3	
MEA4	A4 + C5		MEA8	A2 + C4	

a Active material loading in mg cm^−2^.

Ag-Mn_*x*_O_*y*_/C was used as the cathode or ORR catalyst, and Pd_85_Ni_10_Bi_5_/C as the anode or EOR catalyst. In addition, a commercial Pt/C catalyst was also used for the cathode when the layer thickness of the anode was varied. Catalyst coated substrates (CCSs) were thus manufactured, by spraying catalyst inks on carbon paper (for the cathodes) and on carbon cloth (for the anodes) with an ultrasonic spraycoater (Sonotech ExactaCoat OP3 from SonoTek Corporation, Milton, NY, USA). The thickness and homogeneity of the catalyst layer can be controlled by using the same settings as the spray path and height of the nozzle.^55^ The catalyst inks for all types of catalyst consisted of isopropanol : water (7 : 3) and an ion-exchange ionomer (30 wt% of the amount of catalyst). The Nafion™-ionomer can be used despite alkaline conditions, as shown by Hwang *et al.*^56^ for a direct formate fuel cell, because it serves as a binder and influences the stability. For the cathodes, as well as the anodes, different catalytic active material loadings of 0.25, 0.5, 0.75 and 1 mg cm^−2^ for the determination of the layer thickness influence were achieved. The loading on the anode of Pd_85_Ni_10_Bi_5_/C was 0.5 mg cm^−2^ as the active catalytic material loading on the cathode was varied, whereas the loading of the Pt/C catalyst was 0.5 mg cm^−2^ as the loading on the anode was modified.

The electrodes for the half-cell measurements (Ag-Mn_*x*_O_*y*_/C and Pd_85_Ni_10_Bi_5_/C) were produced by punching out a cyclic sample with a radius of 1.5 cm (active area: 1 cm^2^) from the CCS. The MEAs for the single cell tests were fabricated by assembling the prepared electrodes (active area: 4 cm^2^) and the pre-treated CS/N-rGONRs anion exchange membrane^14^ together. The membrane was pre-treated by placing it in 1 M KOH for 24 h, followed by washing with ultrapure water.

### Scanning electron microscopy

The layer thickness determination was performed with scanning electron microscopy (SEM). The electrodes were thus cut with a sharp scalpel on a hard flat surface to make the cross-section visible. The samples were subsequently adhered to an aluminium SEM holder using conductive carbon tape. The SEM images were taken with a Zeiss ULTRA plus using SE2 and Inlens detectors at 2 kV or 5 kV at WD 5.5 mm.

### Electrochemical half-cell characterization

**Fig. 1:**
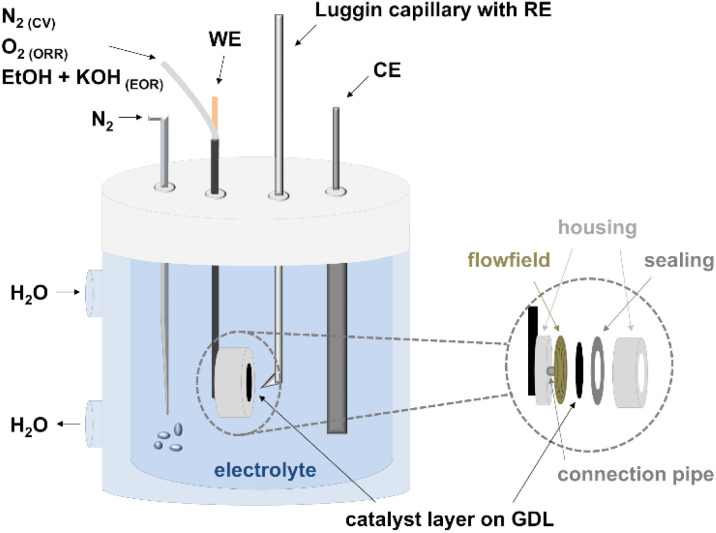
Experimental setup for the half-cell measurements (CV: cyclic voltammetry, ORR: oxygen reduction reaction, EOR: ethanol oxidation reaction, WE: working electrode, CE: counter electrode, RE: reference electrode, and GDL: gas diffusion layer).

The prepared anodes (A1–A4) and cathodes (C1–C4) were first electrochemically characterized in half-cell configuration with cyclic voltammetry (CV) and polarization curve measurements to determine the oxidation and reduction processes of the metals, the ECSA, and the activity towards ORR and EOR. A Zahner IM6ex potentiostat coupled with a PP240 power-potentiostat (Zahner-elektrik GmbH & Co. KG, Kronach-Gundelsdorf, Germany) was used for the measurements. In general, all the measurements were conducted in a three-electrode setup, consisting of a reference electrode (reversible hydrogen electrode (RHE) from Hydroflex® gaskatel) in a Luggin capillary, a platinized titanium rod counter electrode (Bank Elektronik–Intelligent Controls GmbH) and a Diskfix working electrode holder (Bank Elektronik–Intelligent Controls GmbH) with the produced CCS, as shown in Fig. 1.

The Diskfix electrode holder was adapted to have a flow field behind the electrode (to purge through it) as shown by Pinaud *et al.*^34^ This resulted in a surface area of 1 cm^2^. Particular attention was paid to the arrangement of the electrodes in the cell. The counter electrode was placed directly opposite the working electrode and the tip of the Luggin capillary at a distance of 1–2 mm and in such a way that it was not shielding.^34^ The electrochemical testing protocol for the anodes and cathodes is listed in Table 2 and explained in the next sections.

**Table 2:** Electrochemical testing protocol for the catalyst evaluation toward ORR and EOR activity in the half-cell in 5 M KOH^a^

	Step	Technique		Parameters	
				Cathodes (C1–C4)	Anodes (A1–A4)
1	Cleaning	CV	Gas purge	N_2_	N_2_
			Potential limits	0.1–1.0 V *vs.* RHE	0.05–1.50 V *vs.* RHE
			Scan rate	100 mV s^−1^	100 mV s^−1^
			Number of cycles	50	10
			Temperature	RT	RT
2	Ox. and red. processes	CV	Gas purge	N_2_	N_2_
			Potential limits	0.1–1.0 V *vs.* RHE	0.05–1.50 V *vs.* RHE
			Scan rate	10 mV s^−1^	10 mV s^−1^
			Number of cycles	3	3
			Temperature	RT	RT
	ECSA	CV	Gas purge	—	N_2_
			Potential limits	—	0.05–1.20 V *vs.* RHE
			Scan rate	—	10 mV s^−1^
			Number of cycles	—	3
			Temperature	—	RT
3	ORR (cathodes) EOR (anodes)	OCP	Gas or liquid purge	O_2_	3 M EtOH + 5 M KOH
			Time	10 min	10 min
			Temperature	RT, 60 °C, 80 °C	RT, 60 °C, 80 °C
		Polarization curve + *iR* corr.	Potential steps	1/0.97/0.95/0.925/0.9/0.85/0.8/0.75/0.7/0.65/0.6/0.5/0.4/0.3/0.2/0.1	0.1/0.2/0.225/0.25/0.275/0.3/0.35/0.4/0.5/0.6/0.7/0.8/0.9/1/1.1/1.2
			Hold time	10 s	10 s
			EIS frequency range	1–50 kHz	1–50 kHz
			EIS amplitude	5% of voltage	5% of voltage
			*iR*-compensation	100% post correction	100% post correction

a CV = cyclic voltammograms; OCP = open circuit potential.

#### Cathodes

First, the cyclic voltammetry measurements were conducted in 5 M KOH solution at RT. Therefore, cleaning cycles, with N_2_-purging, in a potential range of 0.1–1.0 V *vs.* RHE with a scan rate of 100 mV s^−1^ were performed after flushing the solution for 1 h with N_2_ (step 1). After stabilization base CVs (step 2) were recorded in the same potential range with a scan rate of 10 mV s^−1^. For the evaluation of the ORR activity a polarization measurement routine with the supply of oxygen (25 mL min^−1^) was used. After measuring the OCP, the cell potential was reduced stepwise, with a holding time of 10 s and the current was measured.^34^ In addition, impedance spectra were collected at each specified point in order to perform an *iR* correction.^34,37^ It is important to do this at every point; otherwise errors due to high currents will have a significant impact. The measured current was averaged over the last 5 s of each step and post *iR*-compensated with the measured resistance (intercept on the real-axis).^34,57,58^

This protocol (step 3 in Table 2) was performed at three different temperatures, namely RT (condition I), 60 °C (condition II) and 80 °C (condition III).

#### Anodes

First, the CV measurements were conducted in de-aerated KOH solution. The cleaning cycles were performed with a scan rate of 100 mV s^−1^ in a potential range of 0.05–1.50 V *vs.* RHE to remove impurities from the surface of the electrode. Then, CVs in the same range with a scan rate of 10 mV s^−1^ were measured to see all reduction and oxidation processes of the three metals and subsequently for the determination of the ECSA between 0.05 and 1.20 V *vs.* RHE. The evaluation of the EOR activity (mixture of 5 M KOH and 3 M EtOH with a flow rate of 5 mL min^−1^) for the anodes was comparable to the ORR activity determination for the cathodes as described before. The OCP was determined, and the cell potential was then increased stepwise and *iR*-compensated, while the current was measured. This testing protocol was again performed at different temperatures, RT (condition I), 60 °C (condition II) and 80 °C (condition III).

### Single cell tests

The single cell tests (MEA1–MEA8) were performed with an alkaline DEFC test rig and an in-house fabricated cell, as described in previous work.^19^ The same potentiostat-setup as for the half-cell tests was used to record the polarization curves of the single cells. The cell break-in was performed at 0.4 V for 20 minutes. The polarization curves were recorded in galvanostatic mode, meaning that the current was increased stepwise and the corresponding cell voltage was measured (hold time: 30 s). By multiplying the current density with the cell voltage, the power density was calculated for plotting the power density curve.

All single cell tests were conducted with a cathodic gas flow rate of 25 mL min^−1^ and an anodic fuel and electrolyte flow rate of 5 mL min^−1^. At the cathode either pure or humidified oxygen and at the anode a mixture of 3 M EtOH and 5 M KOH were utilized as fuels, as Abdullah *et al.*^24^ showed with their model that this ratio is optimal. The measurement series included different temperatures for the evaluation of the influence: RT (condition I), 60 °C (condition II) and 80 °C (condition III).

Electrochemical impedance spectra (EIS) were recorded in the single cell configuration under condition III. The spectra were recorded after stabilization for 5 minutes to ensure a steady state, between 50 kHz and 0.1 Hz at 440 mA with a 10% amplitude of the operating point. For evaluation and fitting ZView® software (Scribner Associates Inc., Southern Pines, NC, USA) was used. The equivalent circuit model shown in previous work^19^ was used for the evaluation.

#### Fourier-transform infrared spectroscopy

Determining the ethanol consumption was performed by Fourier-transform infrared spectroscopy.^41,42,44^ A sample of the anodic fuel outlet was taken during the break-in procedure of the cell, since the same voltage of 0.4 V was used for all electrodes. To prevent contamination or a change in the composition of the sample, it was immediately sealed airtight and frozen. These were defrosted for the measurement using an IR spectrometer (Alpha II, Bruker) and the software from OPUS (V 8.1, Bruker Optik GmbH). The absorbance measurements were performed within the 400–4000 cm^−1^ range, with the air spectrum subtraction performed as a background. Visualisation and peak integration of the recorded infrared spectra for the determination of EtOH content were performed using F. Menges “Spectragryph—optical spectroscopy software” (Version 1.2.15). A calibration line of EtOH (3.0, 2.0, 1.5 and 1.0 M) in 5 M KOH was prepared for the quantitative analysis to determine the EtOH concentration.

## Results and discussion

The prepared electrodes (anodes and cathodes) were physicochemically analyzed with SEM to determine the layer thickness. For the electrochemical evaluation of the activity (*e.g.* ECSA for the anodes) and the performance towards the EOR and the ORR, half-cell measurements without external influencing factors were performed. Finally, single cell measurements in an in-house fabricated single cell were made to investigate the influence of factors such as the membrane or ethanol crossover. In addition, EIS measurements were conducted to determine the effect of layer thickness on resistance in the half-cell, as well as in the single cell. IR of the anodic fuel outlet was used to determine the ethanol consumption, the relation to the power density and the estimation of the ethanol crossover.

### Layer thickness determination with scanning electron microscopy

**Fig. 2:**
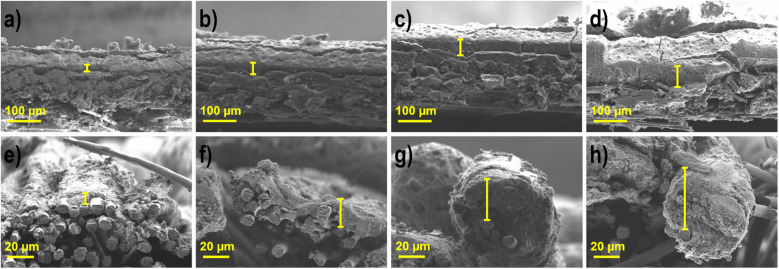
SEM images of the cathodes and anodes with varying active catalytic material loadings (a) C1, (b) C2, (c) C3, (d) C4, (e) A1, (f) A2, (g) A3, and (h) A4.

Determination of the layer thickness of the produced CCS anodes (A1–A4) and cathodes (C1–C4) was performed with SEM analysis, as seen in Fig. 2 and Table 3.

**Table 3:** Layer thickness of the electrodes determined by SEM in μm

Electrodes	Cathodes Ag-Mn_*x*_O_*y*_/C				Anodes Pd_85_Ni_10_Bi_5_/C			
	C1	C2	C3	C4	A1	A2	A3	A4
Thickness (μm)	14 ± 3	29 ± 5	43 ± 7	57 ± 6	7 ± 3	18 ± 5	33 ± 7	46 ± 6

A clear difference in the CCS can be seen in the SEM images, since different gas diffusion layers (GDLs) are used at the cathode and anode, carbon paper on the cathode due to the gas supply and carbon cloth (more hydrophilic) on the anode due to the liquid fuel.^21,59,60^ The layer dimensions become thicker for both types of electrodes; however, as the active material loading increases, the anodes with the same loading as the cathodes, are always somewhat thinner (7–10 μm), which is due to the different catalyst types and the differently used substrate. The woven surface form of carbon cloth is more permeable than that of carbon paper, and thus the active catalyst layer can also be located in the upper part under the threads of carbon cloth. This is also evident when looking at the factors of enlargement of the anodes in comparison to the cathodes. In the case of cathodes, the increase in loading in relation to the previous one occurs at the following intervals: 2, 1.5 and 1.3, and for the layer thickness 2.1, 1.5 and 1.3. The enlargement in the loading is the same for the anodes, but the layer thickness increases by the factors 2.6, 1.8 and 1.4. Thus, the space under the surface of the anode must first be filled, and as the thickness increases, the factors in comparison with the cathodes become nearly equal. These results are in good agreement with the literature. Grandi *et al.*^31^ showed that the layer thickness is reduced by a factor of 2.7, when simultaneously reducing the loading by a factor of 2.5.

### Electrochemical half-cell characterization

The prepared cathodes (C1–C4) and anodes (A1–A4) were electrochemically characterized in half-cell configuration with CV to determine the oxidation and reduction processes of the metals and the ECSA and with polarization curve measurements for the evaluation of the activity towards the ORR and EOR. The half-cell measurement arrangement with the use of a catalyst coated GDE to determine the performance has gained great importance, due to the possibility to visualize the behavior under more realistic conditions (high current densities, realistic catalyst layer structures and three phase boundaries) as with standard rotating disk experiments.^32–34,37,38,40^ Moreover, the influence of various factors of the single cell, *e.g.* membranes, can also be excluded.^37^ Thus, it is a convenient, cost-effective and fast way to determine the influence of parameters, such as the layer thickness or loading, as shown by Schmies *et al.*^33^ Therefore, the evaluation of the impact of the layer thickness and the active catalytic material loading was first performed in the half-cell and subsequently in the single cell. The parameters and operating conditions are listed in the experimental section.

#### Cyclic voltammograms of the anode and cathode electrodes

**Fig. 3:**
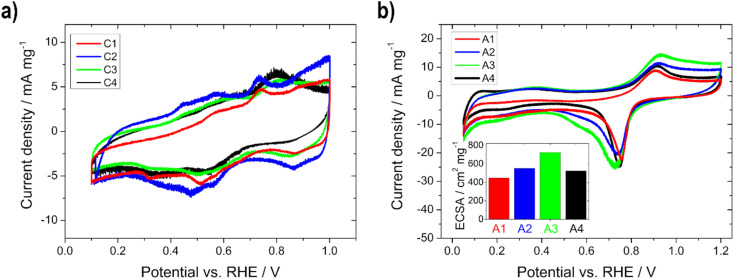
Electrochemical half-cell characterization of (a) the Ag-Mn_*x*_O_*y*_/C (C1–C4) and (b) the Pd_85_Ni_10_Bi_5_/C (A1–A4) electrodes (inset: ECSA) with a scan rate of 10 mV s^−1^ in 5 M KOH at RT.

The electrochemical properties (reduction and oxidation processes) of the Pd_85_Ni_10_Bi_5_/C and the Ag-Mn_*x*_O_*y*_/C CCSs were determined by CV measurements, seen in Fig. 3 and S1.†

In Fig. 3a, the mass normalized CVs of C1–C4 with the several oxidation and reduction steps of manganese oxides can be seen. The Ag redox processes take place in a higher potential range and are therefore not visible. In the anodic scan, Mn(OH)_2_ is oxidized to MnO_2_ (>0.92 V *vs.* RHE) *via* Mn_2_O_3_ (0.4–0.7 V *vs.* RHE) and MnOOH (0.76 V *vs.* RHE), while in cathodic scanning, MnO_2_ is first reduced to MnOOH, followed by Mn_2_O_3_ or Mn_3_O_4_, and further to Mn(OH)_2_. The CVs of C1 and C2, as well as those of C3 and C4, whose loadings are in a comparable range, show a similar profile, respectively. Therefore, we assume that the thickness or the active material loading has an influence on the oxidation and reduction processes and the transformation of the manganese species.^10,12^

In Fig. 3b and S1,† the mass normalized CVs of A1–A4 are shown. In the anodic scan the oxidation of Bi to Bi_2_O_3_ (0.9 V *vs.* RHE) and of Ni(OH)_2_ to NiOOH (1.2–1.5 V *vs.* RHE) are visible. The reduction of NiOOH to Ni(OH)_2_ (1.5–1.2 V *vs.* RHE) and of PdO to Pd (0.9–0.4 V *vs.* RHE) appear in the cathodic scan. No hydrogen ad/absorption (0.05–0.50 V *vs.* RHE) is recognizable due to the alloying of Pd with Bi.^16,17^ The CV in the potential range of 0.05–1.20 V *vs.* RHE was used for the calculation of the ECSA by integrating the Pd reduction peak.^16,17^ The ECSA (Fig. 3b, inset) is increasing with higher active material loading up to the electrode with 0.75 mg cm^−2^ and decreases with higher loading again. Therefore, the following trend can be seen: A1 (450 cm^2^ mg^−1^) < A4 (520 cm^2^ mg^−1^) < A2 (550 cm^2^ mg^−1^) < A3 (720 cm^2^ mg^−1^), meaning that for the thickest electrode the electrochemical active surface area for the reaction is reduced, through reduction of the accessible surface area of the catalyst. To confirm this statement the double-layer capacitance *C*_dl_ at 0.44 V *vs.* RHE (no faradaic processes should occur) for all anode electrodes was estimated. The same trend can be seen: A1 (48 F g^−1^) < A4 (71 F g^−1^) < A2 (108 F g^−1^) < A3 (132 F g^−1^). Since the *C*_dl_ is proportional to the active surface area of all conductive components of the catalyst (support and metal),^61^ the decrease in *C*_dl_ can be described as a decrease in the accessible surface area for the thickest electrode. Li *et al.*^52^ noticed the same phenomenon when the amount of Pd-based catalyst on nickel foam was varied, because the quantity/size of open pores was lowered.

#### ORR and EOR activities of the electrodes

**Fig. 4:**
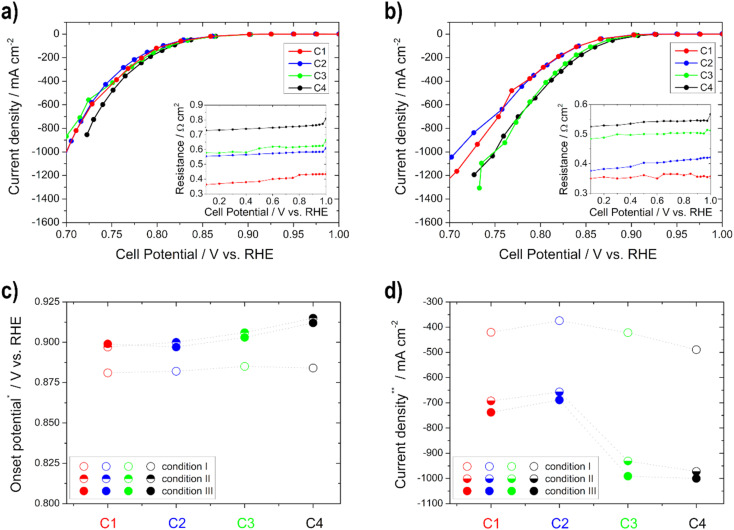
Polarization curves for the determination of the ORR activity for the cathodes C1–C4 under (a) condition I and (b) condition III (resistances of the impedance spectra for *iR*-compensation in the insets); (c) onset potential at −10 mA cm^−2^ and (d) current density at 0.75 V *vs.* RHE for the different conditions.

**Fig. 5:**
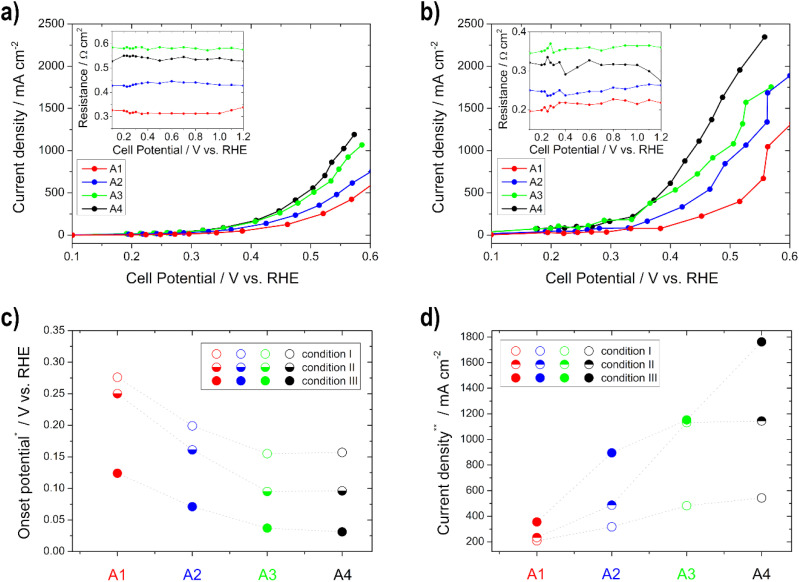
Polarization curve determination of the EOR activity for the anodes A1–A4 under (a) condition I and (b) condition III (resistances of the impedance spectra for *iR*-compensation in the insets); (c) onset potential at 10 mA cm^−2^ and (d) current density at 0.5 V *vs.* RHE for the different conditions.

In Fig. 4, 5, Table S1 and Fig. S2† the results (open circuit and onset potential values, as well as current density) from the polarization curves for the different cathodes (ORR) and anodes (EOR), as well as temperatures are shown.

The activity (higher onset potential and higher current density) of the cathodes improves with an increasing catalyst layer, as seen in Fig. 4, but there is one exception to this in C2, which always achieved a little lower performance or one that was similar to C1 regardless of the temperature. It is remarkable that from condition II onwards with increasing temperature, greater differences in activity can be seen between the individual electrodes. This is related to the fact that diffusion works better at higher temperatures within the electrodes. Therefore, the thicker ones achieve more power due to more active catalyst sites.^35^ Moreover, the electrode resistance values decrease and the current densities at 0.75 V *vs.* RHE get higher with increasing temperature (only slightly between conditions II and III). The onset potential increases with temperature until condition II, and is nearly the same under condition III for all electrodes. This means that in this case an increase in temperature only results in a minimal improvement of the kinetics of the ORR. Current densities between approx. −370 and −490 mA cm^−2^ under condition I and approx. −690 and −1000 mA cm^−2^ under condition III were obtained at 0.75 V *vs.* RHE. Therefore, all of the cathodic electrodes show high values in comparison with the literature values. For example, Ehelebe *et al.*^38^ tested Fe–N–C catalysts for the ORR in alkaline media and achieved current densities of maximum −250 mA cm^−2^ at 0.75 V *vs.* RHE at RT, in 1 M KOH.

The performance (lower OCP, lower onset potential, and higher current density) and the electrode resistance of the various anodes increase for condition I and condition II with thicker layers up to the electrode with a loading of 0.75 mg cm^−2^, and thereafter they do not increase, which is related to the previously determined lower ECSA of the 1 mg cm^−2^ electrode (Fig. 5). This can be explained by the fact that more catalysts and number of active sites are present and thus more reactions can take place.^33,37^ The OCP, the onset, and the electrode resistances decrease and the current density increases with temperature (condition I to condition III) for all electrodes, since the electrode kinetics of the EOR are improved with increasing temperature.^60,62^ The current densities are between approx. 200 and 550 mA cm^−2^ under condition I and approx. 360 and 1770 mA cm^−2^ under condition III, obtained at 0.5 V *vs.* RHE. To the best of our knowledge, there is no literature data about half-cell GDE measurements for the determination of the EOR performance in alkaline media. However, there is literature for the methanol oxidation reaction. Lizcano-Valbuena *et al.*^39^ analyzed the activity of Pt–Ru/C catalysts in acidic media. They reached around 10 mA cm^−2^ by using 3 M methanol fuel.

Based on these half-cell GDE measurements, the previously described advantage over RDE measurements is clearly visible. The CVs are in a similar current range as the RDE measurements from the literature,^12,18^ but higher currents for the ORR can be achieved than with the RDE. Through the use of the gas diffusion layer and the continuous supply of the reactants, the conditions of the fuel cell can be better simulated.^9,32–40^

### Single cell tests

Single cell test characterization of the various electrodes was performed, after assembling them into MEAs. In MEA1 - MEA4 the catalyst loading of the anode and in MEA5–MEA8 of the cathode was varied. In this chapter, the variation of the anode loading and its influence on the ethanol conversion will be discussed first, followed by the variation of the cathode loading and the influence of the catalyst.

#### Quantification of ethanol conversion with infrared spectroscopy

The quantification of the ethanol conversion during the break-in procedure for MEA1–MEA4 was performed with FT-IR by using the anodic fuel outlet. A calibration line with EtOH in 5 M KOH (Fig. S3†) was thus made for this purpose. The pronounced and isolated peak doublet of symmetric and asymmetric C–O stretching of EtOH at 1088 and 1046 cm^−1^ was used for the determination of EtOH consumption.^41,42,44^ Other peaks of the KOH and EtOH mixture, such as the broad high peak between 3600 and 3000 cm^−1^ (O–H stretching), which is overlaid by the band between 3000 and 2800 cm^−1^ (C–H stretching), the water peak 1630 cm^−1^ (bending vibration), and the two smaller peaks at 1600–1200 cm^−1^ (C–H bending vibration) and at 880 cm^−1^ (C–C stretching) would not have been feasible for the determination, due to partial overlapping with possible products, such as acetate (1550 cm^−1^, 1415 cm^−1^, 1348 cm^−1^ and 1018 cm^−1^), carbonate (1390 cm^−1^ and 880 cm^−1^) or acetaldehyde (1640 cm^−1^ and 926 cm^−1^).^41–48,63,64^

**Fig. 6:**
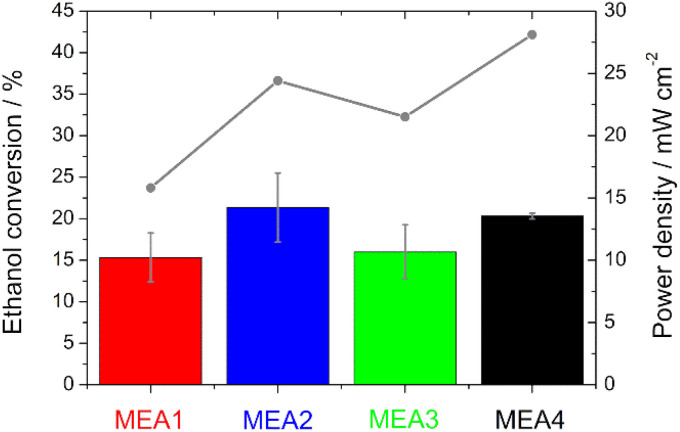
Comparison of the IR analysis (bars) and the power density (grey line) during sampling for the evaluation of the ethanol conversion.

The peak heights of the symmetric and asymmetric C–O stretching double peaks of EtOH are directly proportional to the concentration of EtOH, as well as the area obtained by integration and thus useable for the calibration line and determination (Fig. S3†). Higher EtOH conversion rates were observed for the measurements in which higher power densities were also recorded during sampling (Fig. 6). This means there is definitely a correlation between the power output and EtOH conversion. However, the thinner electrodes show an assumed higher EtOH conversion in comparison with the thicker ones, which is due to the fact that EtOH crossover is facilitated by the use of thinner electrodes.^50^

#### Influence of varying anodic catalyst layer thickness

**Fig. 7:**
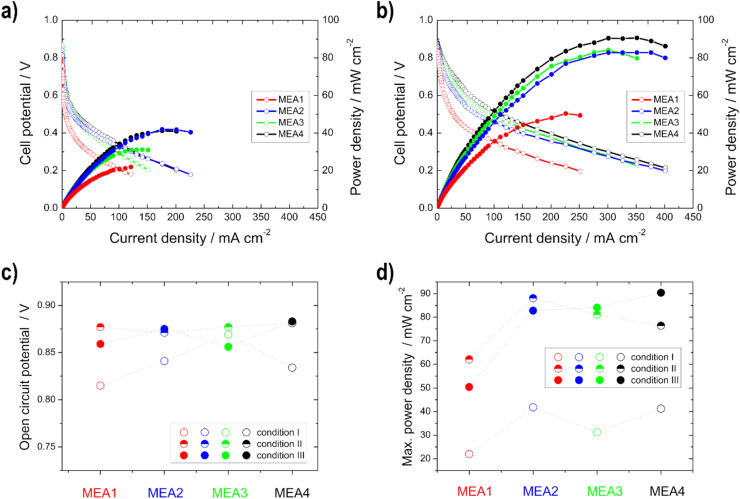
Power density (filled symbols) and polarization curves (unfilled symbols) of the single cell measurements of MEA1–MEA4 under (a) condition I and (b) condition III; (c) open circuit potential and (d) maximum power density for the different conditions.

In Fig. 7 and S4a† the single cell results of the anodic catalyst layer thickness variation (MEA1–MEA4) for the different operation conditions are shown. In theory, the performance should increase with increasing temperature (electrode kinetics, mass transfer properties, and membrane conductivity).^60^ The OCV values increase with increasing temperature but decrease under condition III (80 °C), due to the formation of mixed potentials, when using the Pt/C cathode catalyst. This can be explained by the fact that the possibility for EtOH crossover is increased at higher temperatures and the Pt/C catalyst unfortunately shows EOR activity.^2,11,12,20,50,60,62^

The same trend is evident for the maximum power density values; they decrease for the thinner electrodes under condition III. But there is no loss of power with the thicker electrodes, but rather an increase, since the thickness of the anode electrodes reduces the possibility of an EtOH crossover, as shown with the IR measurements, and thus a loss of power.^50^ This statement is confirmed by the half-cell measurements, as there was no loss of performance or inferior onset potentials at higher temperatures. Moreover, Shao *et al.*^25^ showed for the DMFC that by increasing the electrode thickness the possibility of methanol crossover is reduced.

**Fig. 8:**
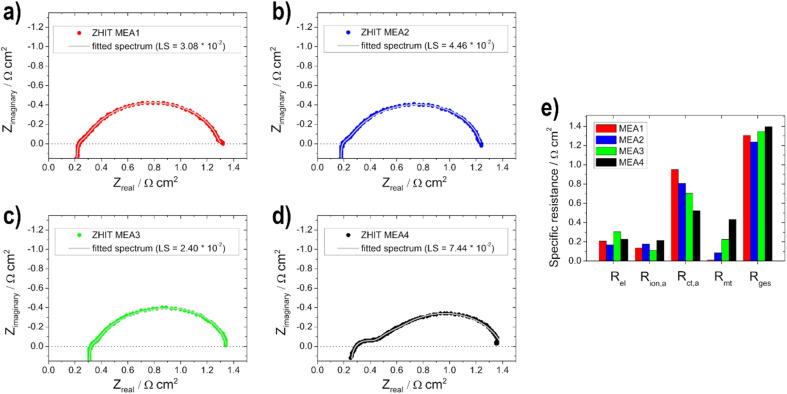
Electrochemical impedance spectra measurements (data: circles—ZHIT algorithm is used and fitted spectrum: line) for (a) MEA1, (b) MEA2, (c) MEA3, (d) MEA4 and (e) comparison of the resistances.

In addition to the polarization curves, EIS measurements (Fig. 8) were conducted to identify the contributions to the voltage loss. The electrolyte resistance *R*_el_ is linked to the ionomer resistance in the anodic catalyst layer *R*_ion,a_ (with opposite behaviour, if the layer thickness is varied). This is due to the presence of KOH in the fuel on the anode side. With varying layer thickness, the catalyst layer is wetted with electrolyte to different levels or not to the extent of the layer, which leads to varying conductivity of the hydroxide ions through the ionomer and membrane. For the two thin electrodes, the addition of the two resistances (*R*_el_ + *R*_ion,a_) gives the lowest total value and increases with the thickness of the layer. *R*_el_ is the lowest for the 0.5 mg cm^2^ electrode (MEA 2). This shows that 0.5 mg cm^−2^ has the best or maximum penetration depth, as also shown by Glass *et al.*^29^ for the DMFC. The charge transport resistance on the anode side *R*_ct,a_ decreases with increasing loading, which is due to the faster or better kinetics of the reactions as determined with the half-cell electrodes for the higher loading and thus more active material on them. The mass transport resistance *R*_mt_ increases as expected with increasing loading due to the thicker layers. The adsorption and formation of intermediates on the catalyst surface can be observed in the low-frequency region of the impedance spectra due to the onset of induction loops, according to Wnuk *et al.*^65^ Looking at the overall resistance *R*_ges_, the 0.5 mg cm^−2^ (MEA 2) fuel cell shows the lowest resistance (1.2 Ω cm^2^) and is therefore favoured, which is in good agreement with the polarization curves. In contrast, the fuel cell with 1 mg cm^−2^ (MEA 4) shows the highest total cell resistance (1.4 Ω cm^2^). This is mainly due to an increased mass transport resistance *R*_mt_ and an increased electrolyte resistance *R*_el_.

#### Influence of varying cathodic catalyst layer thickness

**Fig. 9:**
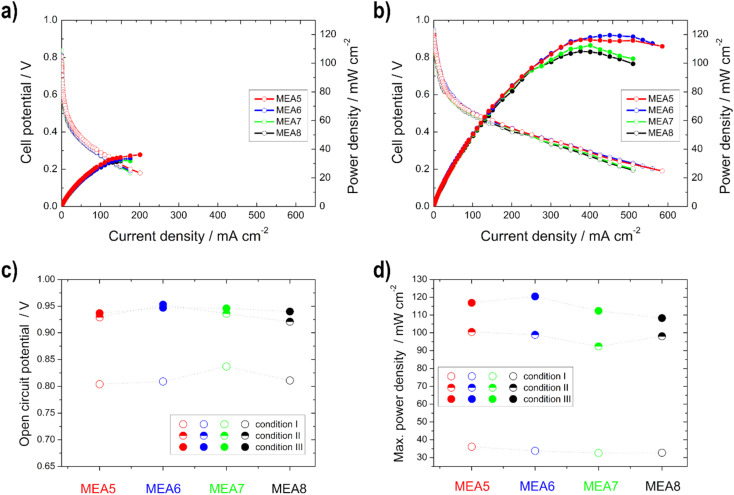
Power density (filled symbols) and polarization curves (unfilled symbols) of the single cell measurements of MEA5–MEA8 under (a) condition I and (b) condition III; (c) open circuit potential and (d) maximum power density for the different conditions.

In contrast to the anodic catalyst layer variation and the half-cell measurements of the cathodes, there is not much difference in the polarization and the power density curves for the different cathode layer modifications in the kinetic region, as seen in Fig. 9 and S4b,† since the limiting reaction side of the alkaline DEFC is the anodic EOR (sluggish kinetics) and not the cathode.^2^

Higher power density values were obtained with the thinner electrodes (measurement MEA5 and MEA6) compared to the thicker ones, especially at higher temperatures. This phenomenon was already described in the literature for Pt/C catalysts in the PEMFC. At higher loading, the more compact catalyst layer prevents gas transport.^37^ Furthermore, it is evident that the difference in the power density curves occurs only in high current density regions, and is thus due to mass-transfer characteristics,^26^ as determined with the higher electrode resistance with the half-cell measurements. Therefore, the better performance of the thinner electrodes can be explained by the better utilization of the catalyst layer and the easier transport of oxygen (shorter pathway) to the active sites.^35^ This is consistent with the literature data from Sievers *et al.*,^35^ which also showed that in contrast to the half-cell measurements, differences in the polarization curves for the single cell occurred in the loading comparison, due to a lower catalyst utilization. The catalyst layer utilization differs in a half and a single cell, since in the case of the cathode measurements, there was contact with oxygen and also with liquid electrolyte during the half-cell measurements, with the result that the reaction took place in the liquid-electrolyte phase. In the case of the single cell measurements, no liquid electrolyte was present on the cathode and thus the reaction took place at the membrane-catalyst layer interface. By using the electrolyte, the electrode is wetted more effectively and the three phase boundary is established more easily and thus, for example, the ion conduction is better.^21,35^

In comparison with the MEA2 measurements (same anode catalyst and loading), higher power density values are achieved for condition III, but not for condition I. This can be explained by the use of the different cathode catalysts, Pt/C was used for MEA1–MEA4 and Ag-Mn_*x*_O_*y*_/C for MEA5–MEA8. At higher temperatures, there is a higher possibility for EtOH crossover through the membrane and in this case the Ag-Mn_*x*_O_*y*_/C catalyst has the clear advantage of being EtOH tolerant compared to the Pt/C catalyst.^2,11,12,60^ Therefore, the intensity to form mixed-potentials is reduced, as shown before with the reduced OCV values, and higher performances at higher temperatures could be achieved with MEAs 5–8 than with MEA2. The OCV values for the single cells with Ag-Mn_*x*_O_*y*_/C as the cathode catalyst, in contrast to the half-cell measurements, increase with increasing temperature due to improved conductivity through the membrane and the improved reaction kinetics at the anode side.^14,60,62^

**Fig. 10:**
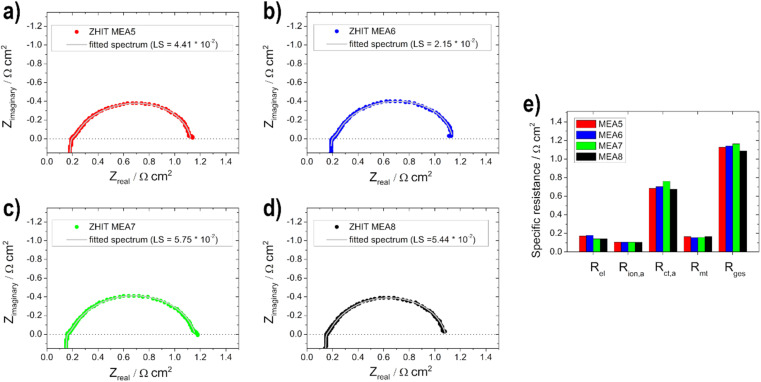
Electrochemical impedance spectra measurements (data: circles—ZHIT algorithm is used and fitted spectrum: line) for (a) MEA5, (b) MEA6, (c) MEA7, (d) MEA8 and (e) comparison of the resistances.

The different EIS spectra shown in Fig. 10a–d of the MEA 5–MEA 8 measurements show that no big difference is noticeable in contrast to the anodic EIS measurements. Overall all of the resistance values show only small changes compared to each other (Fig. 10e).

This is due to the fact that the limiting electrode is not the cathode but the anode, as already mentioned for the polarization curves. Therefore, no significant change in *R*_ion,a_ and *R*_mt_ values can be observed. *R*_el_ decreases slightly with the thicker layer, which, as already shown by Grandi *et al.*^31^ is due to a higher degree of water retention in the catalyst layer and the membrane with increasing catalyst layer thickness. *R*_ct,a_ increases with the thicker cathode layer up to an active material loading of 0.75 mg cm^−2^ and then decreases again; we assume that this effect is due to poorer conduction of OH^−^ ions.^22^ The total cell resistance for all electrodes used is in a range between 1.1 and 1.2 Ω cm^2^.

The open circuit voltage (OCV) as well as the maximum power density values for all single cell measurements can be found in Table 4. A high maximum power density (for this alkaline DEFC system) of 120.5 mW cm^−2^ could be achieved with self-produced catalysts and membranes. An *et al.*^53^ achieved 115 mW cm^−2^ using 5 M KOH, 3 M EtOH and O_2_ at 90 °C. PdNi/C was utilized as the anode catalyst, but the membrane and cathode catalyst were commercial and the loadings were higher. Li *et al.*^52^ achieved 130 mW cm^−2^ at 80 °C with a comparable MEA as that by An *et al.*^53^

**Table 4:** Results of the single cell tests for conditions I, II and III^a^

	OCV/V			Maximum power density/mW cm^−2^		
	I	II	III	I	II	III
MEA1	0.815	0.877	0.859	22.0	62.1	50.4
MEA2	0.841	0.871	0.875	41.8	88.1	82.8
MEA3	0.869	0.877	0.856	31.2	81.1	84.1
MEA4	0.834	0.881	0.883	41.2	76.4	90.4
MEA5	0.804	0.929	0.937	36.1	100.5	116.9
MEA6	0.809	0.953	0.947	33.8	98.9	120.5
MEA7	0.837	0.936	0.946	32.6	92.4	112.4
MEA8	0.811	0.921	0.940	32.7	98.1	108.3

a OCV = open circuit voltage.

It can be concluded as a result that when considering cost and environmental issues, the optimum loading at the anode and cathode for the catalysts used is 0.5 mg cm^−2^ and 0.25 mg cm^−2^, respectively.

Thus, in summary, a similar observation for the single cell measurements can be made as for the half-cell measurements in the context of performance increase on the basis that the higher the temperature, the higher the performance of the cell. This means that the results of the half-cell GDE on the anode side are transferable to the performance of the alkaline DEFC. Other factors, however, such as EtOH crossover or membrane conductivity and also the absence of an electrolyte on the cathode side, as shown, play an important role in the single cell.

## Conclusions

In this study, the effect of the electrode material layer thickness on the performance of the EOR and ORR in an alkaline DEFC was investigated. In addition, the resistance of the electrodes was determined using half-cell and single-cell measurements in conjunction with EIS as well as ECSA and electrode activities. It was found that the half-cell performance increases with higher catalyst content. Furthermore, it has been demonstrated that with GDEs in half-cell configuration much higher currents can be achieved for the ORR than with RDE measurements. An important issue here is that the measurements on single cells also allowed us to evaluate the influence of the membrane or crossover, which was also found to be dependent on the layer thickness. The influence of the film thickness on the resistance was determined with the EIS measurements. For the cathode electrodes, increasing film thickness resulted in an increase in electrode resistance (half-cell) and a decrease in electrolyte resistance due to water retention (single cell). In the single cell, the charge transfer resistance decreases and the mass transfer resistance for the anodes increases with increasing thickness, which is due to the longer paths and penetration depth. In the half cell, the electrode resistance has a relationship with the ECSA, and the higher the ECSA, the higher the resistance. The optimal loading for the anode was determined to be 0.5 mg cm^−2^ and for the cathode to be 0.25 mg cm^−2^ while the highest maximum power density values (∼120 mW cm^−2^) were achieved. This study paves the way for further improvement of MEAs (GDLs) and commercialization of alkaline DEFCs.

## Author contributions

Conceptualization, M.R.; methodology, M.R., S.W., K.M., A.B., B.G., S.G. and V.H.; validation, V.H., B.G. and S.G.; formal analysis, M.R., S.W., K.M., A.B. and B.G.; investigation, M.R., S.W., A.B. and B.G.; resources, V.H., B.G. and S.G.; data curation, M.R., A.B. and B.G.; writing—original draft preparation, M.R. and S.W.; writing—review and editing, V.H., B.G., S.G., M.R., S.W. and K.M.; visualization, M.R.; supervision, V.H., B.G. and S.G.; project administration, V.H., B.G. and S.G.; funding acquisition, V.H., B.G. and M.R.

## Conflicts of interest

There are no conflicts to declare.

## Supplementary Material

SE-007-D2SE01729F-s001
