# Supplementary material for: Influence of the electrocatalyst layer thickness on alkaline DEFC performance
Source: Sustain Energy Fuels. 2023 Jan 27;7(4):1093–106. doi: 10.1039/d2se01729f (PMC9926948; doi:10.1039/d2se01729f)
Supplement: SE-007-D2SE01729F-s001 [file SE-007-D2SE01729F-s001.pdf]

Electronic Supplementary Material (ESI) for Sustainable Energy & Fuels.
This journal is © The Royal Society of Chemistry 2023

SUPPLEMENTARY MATERIAL

Influence of the electrocatalyst layer thickness on alkaline DEFC performance

Michaela Roschger [a], Sigrid Wolf [a], Kurt Mayer [a], Andreas Billiani [a], Boštjan Genorio [b], Selestina Gorgieva [c], and Viktor Hacker [a]

[a]Institute of Chemical Engineering and Environmental Technology, Graz University of Technology, Inffeldgasse 25/C, 8010 Graz, Austria

[b]Faculty of Chemistry and Chemical Technology, University of Ljubljana, Večna pot 113, 1000 Ljubljana, Slovenia

[c]Faculty of Mechanical Engineering, University of Maribor, Smetanova 17, 2000 Maribor, Slovenia

Corresponding author:

Michaela Roschger
Graz University of Technology
Institute of Chemical Engineering and Environmental Technology
Inffeldgasse 25/C, 8010 Graz, Austria
E-mail: michaela.roschger@tugraz.at

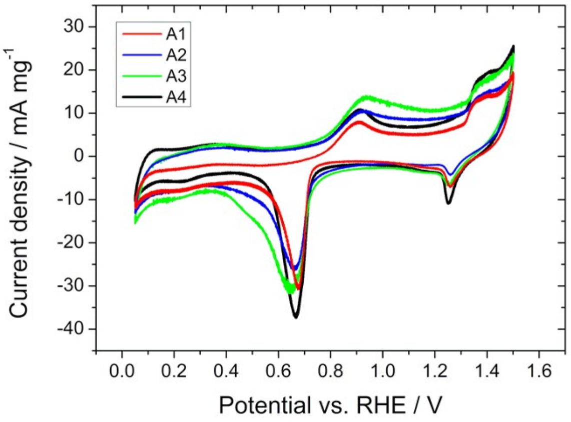

Figure S1: Electrochemical half-cell characterization of the $Pd_{85}Ni_{10}Bi_5/C$ electrodes in a potential range from 0.05 V – 1.50 V vs. RHE with a scan rate of 10 mV s^{-1} in 5 M KOH at RT.

Table S1: Electrochemical characterization results of the cathodes and anodes for condition I, II and III.

	**OCP / V vs. RHE**			**E_{onset}[a] / V vs. RHE**			**j[c] / mA cm^{-2}**			**R / Ω cm^2**		
	**I**	**II**	**III**	**I**	**II**	**III**	**I**	**II**	**III**	**I**	**II**	**III**
**C1**	0.988	0.984	0.980	0.881	0.897	0.899	-420.4	-692.9	-737.9	0.43-0.36	0.34-0.32	0.36-0.35
**C2**	0.997	1.014	0.978	0.882	0.900	0.897	-374.2	-657.4	-689.1	0.61-0.55	0.51-0.44	0.42-0.38
**C3**	0.989	0.997	0.977	0.885	0.906	0.903	-421.8	-931.0	-991.2	0.66-0.58	0.54-0.49	0.51-0.48
**C4**	0.990	1.014	0.985	0.884	0.915	0.912	-489.5	-972.4	-1000.3	0.81-0.73	0.61-0.57	0.57-0.53
	**OCP / V vs. RHE**			**E_{onset}[b] / V vs. RHE**			**j[d] / mA cm^{-2}**			**R / Ω cm^2**		
	**I**	**II**	**III**	**I**	**II**	**III**	**I**	**II**	**III**	**I**	**II**	**III**
**A1**	0.142	0.116	0.058	0.276	0.250	0.124	208.3	234.2	356.0	0.31-0.33	0.28-0.29	0.2-0.23
**A2**	0.106	0.073	0.029	0.199	0.161	0.071	317.2	487.0	895.8	0.43-0.45	0.31-0.32	0.24-0.28
**A3**	0.093	0.054	0.023	0.155	0.095	0.037	482.4	1131.1	1152.7	0.58-0.59	0.41-0.45	0.35-0.37
**A4**	0.076	0.054	0.022	0.157	0.096	0.031	543.3	1144.0	1762.3	0.53-0.55	0.35-0.39	0.27-0.32

OCP = open circuit potential; E_{onset} = onset potential; R = resistance range; j = current density; [a] at -10 mA cm^{-2}; [b] at 10 mA cm^{-2}; [c] j at 0.75 V vs RHE; [d] j at 0.5 V vs RHE

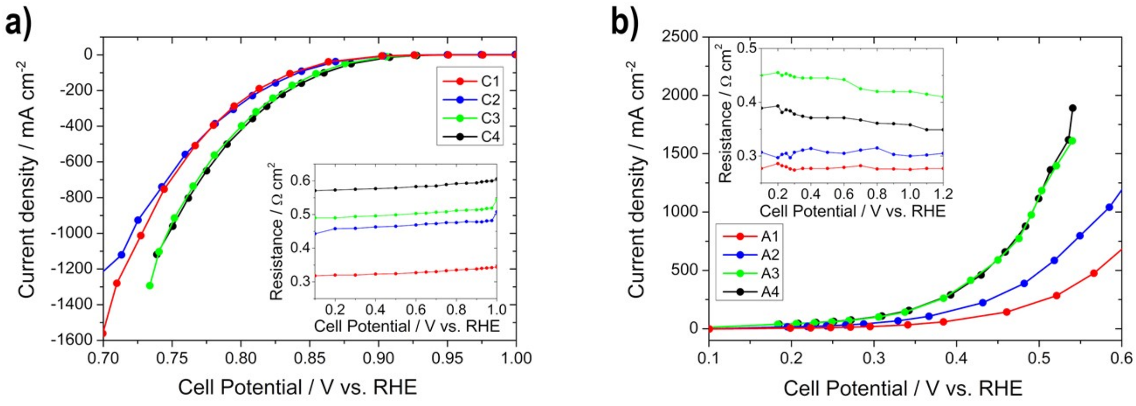

Figure S2: Polarization curves a) for the determination of the ORR activity for the cathodes C1 – C4 and b) for the determination of the EOR activity for the anodes A1 – A4 at condition II (resistances of the impedance spectra for iR-compensation in the insets).

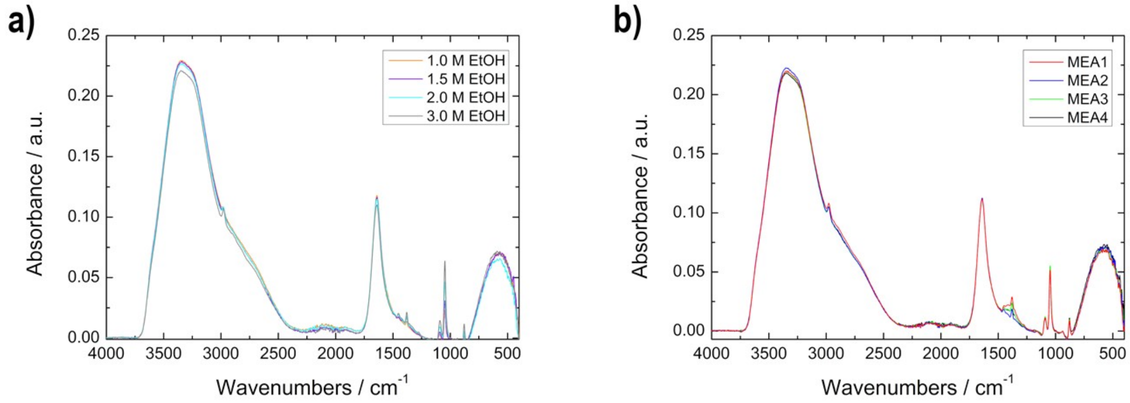

Figure S3: The IR spectrum a) for the EtOH calibration peaks and b) for the evaluation of the consume of ethanol.

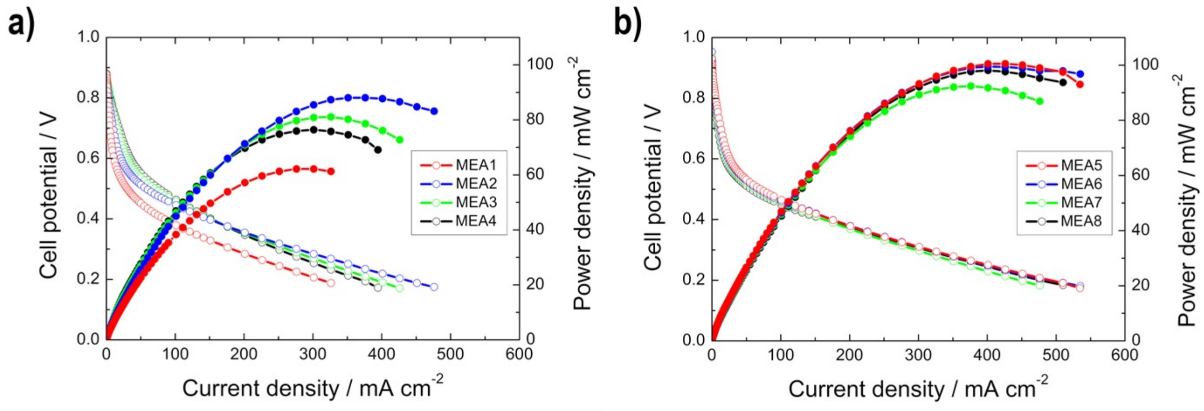

Figure S4: Power density (filled symbols) and polarization curves (unfilled symbols) of the single cell measurements of a) the MEA1 - MEA4 and b) the MEA5 - MEA8 at condition II.
